# Advanced biomaterials for cancer immunotherapy

**DOI:** 10.1038/s41401-020-0372-z

**Published:** 2020-03-02

**Authors:** Fan Yang, Kun Shi, Yan-peng Jia, Ying Hao, Jin-rong Peng, Zhi-yong Qian

**Affiliations:** State Key Laboratory of Biotherapy and Cancer Center, West China Hospital, West China Medical School, Sichuan University, Chengdu, 610041 China

**Keywords:** cancer, immunotherapy, nanobiomaterials, nanoparticles, liposomes, micelles, hydrogel, microneedles, dendritic cells (DCs)

## Abstract

Immunotherapy, as a powerful strategy for cancer treatment, has achieved tremendous efficacy in clinical trials. Despite these advancements, there is much to do in terms of enhancing therapeutic benefits and decreasing the side effects of cancer immunotherapy. Advanced nanobiomaterials, including liposomes, polymers, and silica, play a vital role in the codelivery of drugs and immunomodulators. These nanobiomaterial-based delivery systems could effectively promote antitumor immune responses and simultaneously reduce toxic adverse effects. Furthermore, nanobiomaterials may also combine with each other or with traditional drugs via different mechanisms, thus giving rise to more accurate and efficient tumor treatment. Here, an overview of the latest advancement in these nanobiomaterials used for cancer immunotherapy is given, describing outstanding systems, including lipid-based nanoparticles, polymer-based scaffolds or micelles, inorganic nanosystems, and others.

## Introduction

Cancer immunotherapy is a promising treatment for cancer that aims to provide treatment more accurately and safely than other traditional therapies [1, 2]. Agents are designed to provoke a robust primary and secondary antitumor immune response by repairing or enhancing natural mechanisms that are evaded or damaged during disease progression, thus inhibiting tumor growth and metastasis [3–5].

**Fig. 1:**
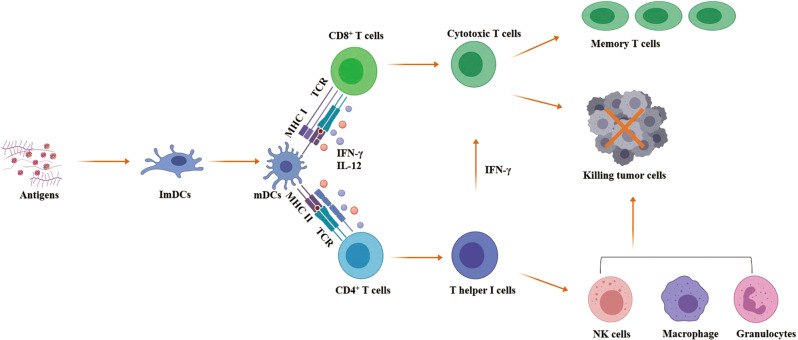
Scheme of the cancer immunotherapy mechanism. After antigens are processed by immature dendritic cells (ImDCs), they are presented to T cells by mature dendritic cells (mDCs) through major histocompatibility complex (MHC) class I or MHC class II complexes binding to CD8^+^ or CD4^+^ T cells, separately. Simultaneously, mDCs also express costimulatory molecules and cytokines such as IFN-γ and IL-12 to synergistically stimulate T cells. CD8^+^ T cells further differentiate into cytotoxic T lymphocytes (CTLs), and CD4^+^ T cells further differentiate into IFN-γ secreting T-helper 1 (Th1) cells to assist in activating CD8 cells and other innate immune cells, such as natural killer (NK) cells, granulocytes or macrophages, to directly kill tumor cells

Approximately a century ago, Coley first used a method to activate the patient’s immune system to help treat tumors [6]. In the immune system, antigen-presenting cells (APCs) continuously eliminate exogenous or endogenous antigens; antigens are taken up and processed to be exposed onto major histocompatibility complexes (MHCs) I or II on the APC surface for further presentation to naive T cells [7, 8]. The three main pathways by which APCs activate T cells are the binding of MHC complexes to T-cell receptors, the presence of costimulatory molecules on the cell surface (CD80 and 86 on APCs binding to CD28 on T cells) and the cytokines that stimulate T cells [9]. T cells can differentiate into two major subpopulations: CD4^+^ T cells, which can further differentiate into T-helper 1 (Th1) and T-helper 2 (Th2) cells, and CD8^+^ T cells, which can further differentiate into cytotoxic T lymphocytes (CTLs) to directly kill tumor cells [9, 10]. Both CD8^+^ T cells and IFN-γ-secreting Th1 CD4^+^ T cells play a vital role in killing tumors [10, 11] (Fig. 1).

In 1986, the US Food and Drug Administration (FDA) approved recombinant versions of the cytokine interferon-α (IFN-α) as the first cancer immunotherapeutic drug for the treatment of hairy cell leukemia; however, IFN-α was replaced because of its short therapeutic duration [12]. Subsequently, recombinant interleukin-2 (IL-2) was approved by the FDA as a cancer immunotherapy drug for the treatment of metastatic renal cancer (in 1992) and metastatic melanoma (in 1998), separately [13]. Although IL-2 initially has a good therapeutic effect in some patients, the use of large doses due to its short half-life results in many immune-related side effects, such as cytokine release syndrome and vascular leakage syndrome [14–16]. After a stagnant phase, sipuleucel-T (an autologous dendritic cell (DC) therapy) as the first cancer therapeutic vaccine was approved by the FDA for prostate cancer, which meant tumor immunotherapy had finally made successful progress in the early 21st century. However, production complexities and other issues hindered the clinical translation of sipuleucel-T [17, 18]. Since the cytotoxic T-lymphocyte antigen-4 (CTLA-4)-targeted checkpoint inhibitor ipilimumab was approved for advanced melanoma in 2011 [19], there has been a shift towards novel immunotherapies, including programmed cell death-1 or its ligand monoclonal antibody (aPD1 or aPDL1) [20] and chimeric antigen receptor (CAR) T-cell therapies [21–23].

Although these treatments have been developed and approved for clinical use and have achieved some efficacy, many problems regarding safety and effectiveness remain to be solved [14, 24, 25]. In terms of safety, some immunotherapeutic drugs require a large dose for their short half-life, which causes autoimmune side effects in some patients. For example, two syndromes (cytokine release syndrome and vascular leakage syndrome) caused by IL-2 lead to severe and even lethal systemic inflammatory reactions in some patients [25]. In terms of efficacy, current immunotherapy is only effective in some patients, and most immunotherapy is initially used only to treat hematological tumors. Only a few immunotherapies for the treatment of solid tumors are approved because solid tumors have a complex tumor microenvironment (TME) that is a difficult barrier to break through [26].

**Fig. 2:**
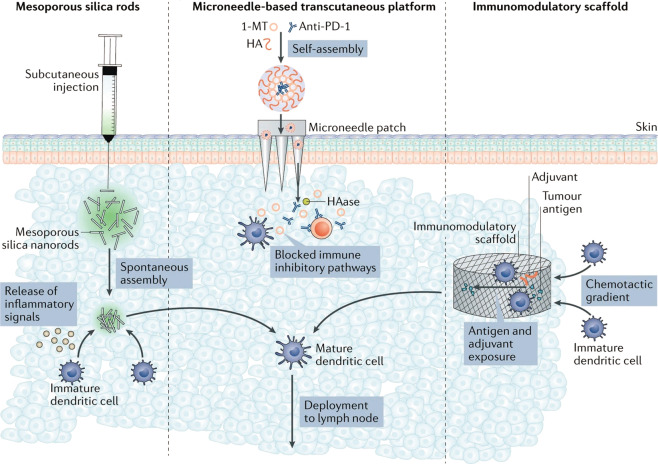
Different biomaterials for cancer immunotherapy. Reprinted with permission from [2]

To reduce side effects and improve the accuracy of immunotherapy, novel delivery systems need to be manufactured. In recent years, with the development of nanotechnology, an increasing number of delivery systems have been designed for the local and sustained release of immunotherapeutic drugs in vivo [27–29]. Biomaterial-based delivery systems have many advantages in cancer immunotherapy, such as the specific and targeted delivery of biomolecules, high efficacy, low toxicity, and immune-stimulating effects (Table 1) [27, 30, 31]. A great variety of advanced biomaterials can be used for cancer immunotherapy, including liposomes, polymers, silica, and so on (Fig. 2) [2, 32, 33]. Different biomaterials use various means and technologies to play an important role in cancer prevention [34–38]. To achieve precise antitumor effects, these advanced biomaterials with different functions can be used to deliver immunopharmaceuticals to organs or tissues (such as the mucosa or skin) that are rich in immune cells by different routes of administration (for instance, intranasally [39], orally [40], and subcutaneously [41]).

**Table 1:** Characteristics of selected delivery strategies for cancer immunotherapies

Delivery technology	Classes of immunotherapy	Advantages	Limitations
In vivo nanoparticle delivery to immune cells	• Cytokines • Checkpoint inhibitors • Agonistic antibodies • Engineered T cells	• Surface functionalization with targeting agents • Localized delivery • Cargo protection	• Premature drug release • Nanoparticle stability • Delivery to off-target clearance organs • Systemic toxicity
Ex vivo T-cell functionalization with nanoparticles	• Cytokines • Vaccines • Engineered T cells	• Innate tumor infiltration • Improved drug delivery • Can be engineered ex vivo or in vivo	• Long production time • Short drug release profiles • Cell death after administration • Complex manufacturing
Controlled release systems	• Cytokines • Checkpoint inhibitors • Agonistic antibodies	• Extended therapy timeline • Cargo protection • Low required doses • Localized delivery following intravenous injection	• Difficult to control release profiles • Toxicities from off-target release • Potentially require surgical implantation • Acidification can degrade cargo
Biomaterial implant scaffolds	• Cytokines • Vaccines • Engineered T cells	• In situ dendritic cell activation • Delivery of dendritic cell attractants • Implant functionalization with antigen • Controlled release profiles • Provides physical structure for cells	• Potential toxicity from the implant material • Need to define specific antigens • Potential rejection of loaded adjuvant • Requires surgery
Injectable biomaterial scaffolds	• Cytokines • Checkpoint inhibitors • Neoantigens	• Minimally invasive • No surgery required • Controlled release of loaded cargo • Delivery directly to the tumor	• Early stages of development • Requires extensive characterization for biodegradation profile • May require large gauge needle
Transdermal delivery systems	• Checkpoint inhibitors • Neoantigens	• Sustained release • Low required doses • Local delivery directly to the tumor • Minimally invasive • Bio-responsive	• Small treatment area • Bioavailability and biocompatibility are unknown • Can be used only for tumors close to the skin • Complex manufacturing

Reprinted with permission from [2]

In this review, we first summarize several major immunotherapeutic strategies and discuss their strengths and weaknesses. We then describe a range of advanced nanobiomaterials that have been used for tumor immunotherapy to enhance efficacy and/or reduce side effects and evaluate the clinical or preclinical impact of these strategies.

## Main classifications of cancer immunotherapy

This article mainly focuses on advanced biomaterials for the following four categories: cytokines, immunological checkpoint inhibitors, engineered T cells, and cancer vaccines. In this section, we outline the four immunotherapies and analyze the options that can be used to address their shortcomings.

### Cytokines

As recombinant IFN-α was approved for the treatment of hairy cell leukemia, cytokines were the first class of immunotherapy for clinical use [12]. After cytokines are injected into the body, immune cells can be directly activated to generate an immune response. The current cytokines used in immunotherapy mainly include interferon, interleukin, and granulocyte macrophage colony-stimulating factor (GM-CSF) [14]. Usually, when the body is infected by pathogenic microorganisms, interferon is produced to induce the activation and maturation of macrophages, lymphocytes, DCs, and other immune cells [42]. In addition, interferon can also inhibit angiogenesis at the tumor site [43]. Interleukins promote the activation and differentiation of CD4^+^ T cells, CD8^+^ cells, and B cells to promote innate and adaptive immune responses [44–46]. GM-CSF is a cytokine that promotes the differentiation of bone marrow cells; it plays a key role in the activation of DCs and the priming of antitumor CTLs [47]. Both granulocyte colony-stimulating factor (G-CSF) and GM-CSF were approved for use in neutropenia [48]. Although cytokines have a certain effect, due to the short half-life of cytokines, large doses are needed to achieve better therapeutic effects, thus leading to cytokine release syndrome [14]. In addition, cytokines may promote regulatory T-cell growth while inducing stimulated T-cell death, which leads to the emergence of autoimmune diseases [24]. Researchers are currently trying to combine several cytokines or combine cytokines with chemotherapy or other immunotherapies to reduce the dose used and thereby avoid the side effects caused by high doses [43].

### Immunological checkpoint inhibitors

Immunological checkpoint inhibitors are by far the most studied immunotherapies, and the most commonly used inhibitors are PD-1/PD-L1 (programmed death receptors 1/programmed death receptor-ligand 1) blockade and CTLA-4 inhibition. Immune checkpoints are inhibitory pathways in the immune system that maintain self-tolerance and regulate the physiological immune balance [49]. Activated T cells express PD-1 to recognize and remove abnormal or cancerous cells [50]. However, tumor cells inactivate the T cells that recognize tumor antigens by expressing PD-1 ligands that bind to PD-1, thereby evading immune system attacks [51]. Therefore, tumor cell death can be induced by blocking inhibitors of PD-1 or its ligand. CTLA-4 is another immune checkpoint that reduces T-cell activation and promotes tumor progression by binding to its ligands (CD80 and CD86) [52]. Inhibitors against CTLA-4 and its ligands block their interactions to increase T-cell activity and thereby clear tumors.

In the past few years, PD-1, PD-L1, or CTLA-4 checkpoint blockade strategies have achieved encouraging clinical results. More than five checkpoint inhibitors have been approved for different tumors [53]. However, it is disappointing that there are many limitations to their use. As described above, the systemic use of immunological checkpoint inhibitors can cause damage to many normal organs [54, 55]. In addition, not all patients can benefit from checkpoint inhibitors, and studies on the reactivity to checkpoint inhibitors are also underway [56, 57]. Furthermore, the immunosuppressive microenvironment of different tumors varies, and new strategies are needed to achieve precise treatment of tumors [58]. With the development of nanotechnology, these limitations may be addressed by using advanced biomaterials to achieve the precise delivery of drugs at the tumor site.

### Engineered T cells

Engineering T cells mainly include CAR T cells and T-cell receptor T cells (TCR T cells). In recent years, CAR T cells have achieved great clinical success and received FDA approval. In CAR T-cell therapy, T cells collected from the peripheral blood of a patient are engineered in vitro to express CARs that specifically recognize tumor antigens and then are reinjected into the same patient to recognize and kill tumors; [59] these T cells can maintain activity for a long time in the body [60]. However, CAR T-cell therapy is time-consuming, expensive, and technically demanding, which limits its widespread use [61]. In 2017, two CAR T-cell therapies targeting CD-19 were approved for the treatment of lymphoma [62, 63]. B-cell leukemias and lymphomas highly express CD-19 molecules. Moreover, normal cells expressing CD-19 have only B-cell lineages. Therefore, the main side effect of CAR T cells targeting CD-19 is B-cell hypoplasia, which can be alleviated by immunoglobulin replacement therapy [64]. The clinical success of CD-19 CAR T cells has inspired many studies of CAR T-cell therapy for different antigens or combinations of several antigens [65–67]. However, CAR T-cell therapy causes cytokine release syndrome and neurotoxicity [68], and in some cases (especially solid tumors with hash microenvironments), the survival of CAR T cells is affected [69, 70]. Therefore, new biomaterials and techniques are urgently needed to improve the survival of CAR T cells.

TCR T cells are clinically available for hematological and solid cancers. TCRs respond to MHC-presented tumor-associated intracellular antigens, such as neoantigens and cancer-testis antigens [71]. TCR T cells are MHC-dependent immunotherapies. In addition, preclinical studies have shown that the specificity of TCR T cells plays an important role in clinical outcomes; [72] however, the toxicity caused by high-affinity TCR T cells is also difficult to predict [73]. For the above reasons, it is particularly important to develop new technologies and new biomaterials to avoid the toxicity of CAR T cells and TCR T cells while improving their applicability to solid tumors.

### Cancer vaccines

The four main cancer vaccines are DCs, tumor cell lysates (TCLs), nucleic acids, and neoantigens [74]. DC vaccines are a widely studied class of cell-based tumor vaccines in which DCs obtained from patients are stimulated in vitro to express tumor-associated antigens and then directly activate T cells to kill tumors. As mentioned above, one DC vaccine approved for prostate cancer is sipuleucel-T [75]. However, other DC-based vaccines have failed in clinical studies despite their high safety [76]. It is predicted that the therapeutic effect on a tumor can be improved by increasing the expression level of the target antigen on the surface of the DC [77] and promoting the lymph node delivery efficiency of the DC vaccine [78].

Tumor cell lysates can be prepared by two common clinical methods: ultraviolet B ray irradiation or freeze–thaw cycles. Tumor cell lysates contain a variety of tumor-associated antigens, which can avoid ineffective immunization caused by the loss of a single antigen after tumor mutation. In addition, TCLs are suitable for all patients and are not restricted to their HLA type [79]. Clinical experiments have shown that tumor cells can be modified to express more immune-related elements, including interleukins, costimulatory molecules, etc., so that TCLs can produce a stronger antitumor immune response in vivo after injection [74]. However, many TCL-associated vaccines have failed in Phase II or Phase III clinical trials [80], suggesting that tumor lysate vaccines require new techniques and materials to increase their effectiveness.

The nucleic acid vaccine is a promising alternative to traditional vaccines that work by delivering exogenous nucleic acids into target cells [81]. Nucleic acid therapeutic drugs consist of DNAs or RNAs that are taken up and translated into antigenic proteins by APCs, which induce immune responses against the target proteins to kill tumors expressing target antigens [81]. Although DNA-based nucleic acid vaccines have been clinically tested, they have failed due to their intranuclear delivery difficulties and somewhat disappointing immunogenicity [82, 83]. In contrast, RNA-based nucleic acid vaccines can be directly translated into antigenic proteins, which enhance immune efficiency. Furthermore, since RNA is a naturally occurring molecule, its preparation cost is low, and its half-life can be extended by modification. Moreover, RNA is not integrated into the genome as DNA is and therefore does not cause hereditary side effects [84]. However, naked RNA is highly susceptible to degradation by nucleases; therefore, it requires special transfection reagents or delivery techniques to enhance its intracellular delivery [85]. Thus, nucleic acid vaccines can greatly benefit from advanced delivery technologies and materials, which are technical barriers to current nucleic acid vaccines. An effective and safe delivery system is the key to the successful application of nucleic acid vaccines.

Neoantigen vaccines use tumor somatic DNA as antigens to promote the antitumor immune response [86]. These antigens are only expressed in tumor cells and can avoid damage to normal tissues [87]. Advanced materials and delivery systems can improve the stability of these neoantigens and combine multiple vaccine classes to improve the safety and efficacy of cancer vaccines [88–90].

## Novel biomaterials for cancer immunotherapy

### Lipid-based biomaterials

**Fig. 3:**
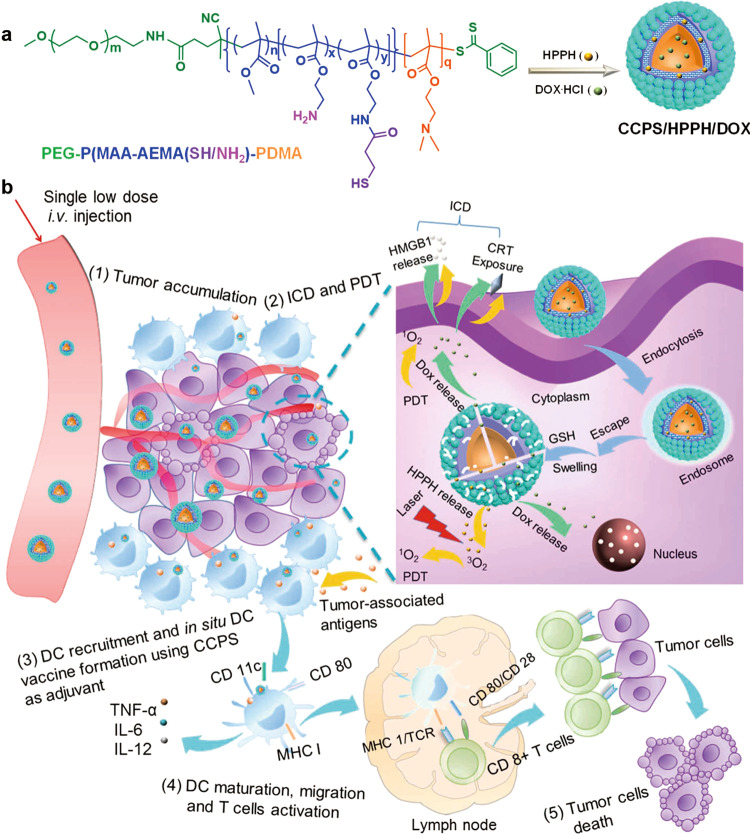
Schematic depiction of an in situ DC vaccine using chimeric cross-linked polymersomes (CCPS) as adjuvants combined with PDT and ICD for the treatment of MC38 colorectal cancer. **a** Process of self-assembled nanoparticle formation. **b** Immune response in vivo after injection of CCPS/HPPH/DOX. Reprinted with permission from [98]

As the most powerful professional APCs, DCs have the potency of integrating both innate and adaptive immunity [91–94]. Antigens must be seized by APCs, especially DCs, to induce a cytotoxic CD8^+^ T-cell (CTL) immune response directed against tumors (Fig. 3) [95–98]. Since cellular DC vaccines require a time-consuming and costly preparation process [99, 100], lipid-based nanobiomaterials such as liposomes have been explored to deliver antigens and adjuvants directly to DCs in vivo [101–103].

#### Liposomes

Liposomes are nanosized bubbles made of a phospholipid bilayer [104, 105], which are the main lipid-based nanobiomaterials. Liposomes have been studied as potential vectors of immunotherapy for many years, including marketed vaccines for influenza (Inflexal^®^), Hepatitis A (Epaxal^®^), and recently, malaria (Mosquirix) for the property of being easily functionalized [106]. Lai et al. synthesized liposomes that were loaded with a melanoma-specific TRP2180-188 peptide and modified with the DC-targeting mannose and immune adjuvant CpG-ODN. These liposomes significantly increase tumor antigen-specific CD8^+^ cytotoxic T cells, and this leads to the inhibition of tumor angiogenesis and tumor cell proliferation [107]. Le Moignic et al. reported a dramatic mRNA delivery system made with cationic liposomes (L), a cationic polymer (P), and mRNA (R) that promoted DC-targeting and exerted cogent antitumor responses [102].

To increase the environmental responsiveness of liposomes, highly pH-sensitive polymers were modified on the surface of liposomes: they promoted the secretion of Th1 cytokines from DCs and enhanced the therapeutic effects of the tumor [108, 109]. Yuba et al. designed a codelivery system to simultaneously load antigen and IFN-γ genes by using pH-sensitive liposome-loaded antigen and lipoplexes containing the IFN-γ gene, which resulted in strong therapeutic effects [110]. Moreover, combining these pH-sensitive liposomes with DC-targeting adjuvants could trigger double stimulation to DCs and promote antitumor immunity [111].

Another study suggested that liposomes that were modified with small molecule inhibitors to prevent TGFβ from binding with the TGFβ receptor and preloaded on T cells could improve the infiltration of T cells into B16F10 melanoma tumors more efficiently [112]. Drugs wrapped by liposomes have a prolonged blood circulation time and reduced adverse effects [113]. In recent years, a cytotoxic T-lymphocyte-associated protein-4 (CTLA-4)-blocking monoclonal antibody had been used in clinical research and achieved some effects [114, 115]. However, its adverse effects were also serious [116]. Nikpoor et al. studied the antitumor effects of liposomes containing a CTLA-4 blockade antibody and proved that the PEGylated liposomal formulation of CTLA-4 could improve the antitumor immune response and therefore merited further human tumor-associated studies [117]. To improve the efficiency of antigen delivery, Miura et al. reported liposomes modified with a KALA peptide (WEAKLAKALAKALAKHLAKALAKALKA), an α-helical cationic peptide, and encapsulated antigens that delivered antigens to the cytoplasm and achieved a more antigen-specific cytotoxic T-lymphocyte response [118].

Recently, many studies proved that combined photothermal immunotherapy could potentiate the antitumor immune response and overcome the limitations of photothermal therapy (PTT) [119, 120]. Li et al. illustrated that fluorophore-loaded liposomes (IR-7-lipo) modified with an immunoadjuvant (HA-CpG) could synergistically activate CD8^+^ T effector cells and alleviate the immunosuppressive TME [121]. Furthermore, researchers prepared liposome-coated gold nanocages containing adjuvant MPLA and melanoma antigen peptide TRP2 and modified them with the DC-specific antibody aCD11c on the surface; they confirmed that these liposomes potentiated the activation and maturation of DCs by the targeting capacity of aCD11c, and the migration of DCs could also be monitored in real time by fluorescence and photoacoustic imaging [122].

#### Lipid/calcium/phosphate (LCP) nanoparticles

LCP nanoparticles (NPs) are made of lipid-coated CaP [123]. Liu et al. combined an anti-CTLA-4 monoclonal antibody with the LCP-based mRNA vaccine encoding tumor antigen MUC1 to DCs to promote antitumor effects [124]. This combination administration significantly enhanced the T-cell immune response and proved that LCP is an effective carrier for delivering tumor-associated RNA. Moreover, an LCP-based cancer cell-mimicry vaccine (αHSP70p-CM-CaP) was reported to kill tumor cells and suppress tumor infiltration [125]. Huo et al. encapsulated sunitinib base into PLGA-PEG-MBA (SUN_b-PM_) polymeric micelles and simultaneously hypodermically injected an LCP-based vaccine encompassing tyrosinase-related protein 2 (Trp2) peptide and CpG-ODN; they proved that SUN_b-PM_ could enhance the effects of immunotherapy for advanced melanoma by modulating the TME [126].

### Polymer-based biomaterials

#### Micelles

When the critical micelle concentration was reached, amphiphilic polymers self-assembled as nanosized particles, namely, micelles [127, 128]. Particles with a smaller size and neutral charge may arrive more easily at the draining lymph nodes and enhance systemic spread [90, 129–132].

To induce systemic toxicity and promote the maturation of DC, Zeng et al. produced an amphiphilic molecule (PSA) by modifying polyethyleneimine (PEI)-2k with stearic acid, and PSA micelles selectively accumulated in the draining lymph nodes rather than the systemic area. Moreover, PSA micelles stimulated the secretion of CCR-7 and the expression of CD86 and MHC-II in draining lymph nodes, and PSA micelles loaded with Trp2 showed significant antitumor effects [133]. Poly(ethylene glycol)-block-poly(L-arginine)-based micelles delivered to a tumor site via enhanced permeability and retention effects suppress tumor progression by targeting macrophages and promoting the generation of NO in the tumor site [134]. Another tumor-associated macrophage (TAMs)-targeted micelle, galactose-functionalized zinc protoporphyrin IX (ZnPP)-grafted poly(*L*-lysine)-b-poly(ethylene glycol) polypeptide micelles (ZnPP PM), was reported, and these micelles could repolarize TAMs to M1 macrophages by producing reactive oxygen species (ROS) [135].

A mixed micellar system loaded with curcumin (CUR) was proven to have good bioavailability and to downregulate proinflammatory cytokines, which provided a new idea for surmounting the development of resistance in endometrial cancer [136]. Another CUR–PEG micelle, a curcumin–polyethylene glycol conjugate, was designed to promote antitumor effects combined with a vaccine, and it significantly increased interferon-γ (IFN-γ) production and reduced immunosuppression in the TME [137]. However, a high concentration of IFN-γ may induce systemic toxicity. Ishii et al. designed polyion complex (PIC) micelles whose viscosity was low at room temperature but increased under physiological conditions and proved that this gel loaded with IL-12 could inhibit tumor growth and decrease side effects [138].

Cui et al. investigated polyplex micelles loaded with genes encoding the tumor-associated antigens SART3, GM-CSF, and CD40L and demonstrated that these micelles when administered subcutaneously may be safe and effective for tumor immunotherapy [139]. Researchers manufactured polymeric hybrid micelles (PHMs) loaded with Trp2 and CpG, and these PHMs consisted of polycaprolactone–polyethylene glycol (PCL–PEG) and 10% (w/w) PCL–PEI and were proven to have the ability to deliver antigen and adjuvant to lymph nodes and stimulate the CTL response, thus effectively inhibiting tumor growth [140]. Luo et al. prepared poly(ethylene glycol)-b-poly(L-lysine)-b-poly(*L*-leucine) (PEG-PLL-PLLeu) micelles coloaded with OVA and STAT3 siRNA and proved that these micelles downregulate the expression of STAT3 and promote the maturation of DCs, thus improving the therapeutic efficacy of tumor vaccines [141]. Moreover, PEG-PLL-PLLeu self-assembled into micelles containing miR-148ai, and OVA reprogrammed the immunosuppression of tumor-associated dendritic cells (TADCs) by inhibiting the DNMT1 gene, which upregulated SOCS1, the suppressor of TLR signaling, and these findings provided a novel idea for antitumor immunotherapy [142]. Li et al. designed carboxylated micelles modified with OVA and toll-like receptor-7 agonist CL264, which showed the ability to deliver antigen and adjuvant to lymph nodes, thus stimulating cellular and humoral antitumor immune responses [143].

#### Nanoparticles

PLGA copolymers are biodegradable aliphatic polyesters made of diverse proportions of lactic and glycolic acids, which are approved by the FDA to be used in the production of surgical sutures and in some drugs to achieve controlled release [144–147]. Because its degradation products can be metabolized normally, PLGA has good biocompatibility, and therefore, many researchers use PLGA to deliver not only antitumor agents or drugs such as paclitaxel [148], 9-nitrocamptothecin [149], and estradiol [150] but also cancer vaccines [151–156].

Researchers have successfully manufactured PLGA NPs loaded with cancer cytomembranes, which can simultaneously deliver tumor-associated antigens and adjuvants so that these NPs can promote the expression of surface biomarkers on DCs. Moreover, this strategy can be used to target the source of cancer cells by an isotypic combining mechanism and is beneficial to antitumor drug delivery [157]. A PLGA-based biomimetic artificial antigen-presenting cell (aAPC) combined with an anti-PD-1 monoclonal antibody has been proven to have synergistic effects on tumor immunotherapy by motivating cytotoxic CD8^+^ T cells and decreasing the inhibition of the tumor immunosuppressive microenvironment [158]. PLGA-based NPs that were coloaded with antigens and MPLA can be fractured in the intracellular milieu and promote antigen retention and IFN-γ secretion simultaneously by enveloping the erythrocyte membrane on the surface [159].

PLGA-based microspheres were found to enable the eradication of prostate carcinoma by codelivering tumor lysates, CpG-ODN, and Poly(I:C), and their capacity to stimulate T cells to produce IFN-γ and granzyme B was significantly enhanced in TRAMP mice [160]. PLGA polymers were also used to codeliver OVA, CpG-ODN, and Poly(I:C); however, the therapeutic effects were weakened, and the production of IFN-γ and the activation of DCs were decreased under chronic stress [161]. Two TLR7/8 agonists have been synthesized and encapsulated in PLGA NPs. This OVA or tumor lysate NP vaccine significantly inhibits tumor growth in B16F10-OVA or renal cell carcinoma by stimulating the CD8^+^ CTL response [162].

To realize image-guided delivery of immunomodulators, IFN-γ and iron oxide nanocubes were coencapsulated in PLGA microspheres. This delivery system could provide a convenient way of delivering drugs to tumor sites after injection and monitoring the distribution of drugs sequentially [163]. A new combination method that uses PLGA for encapsulating a physical mixture of ovalbumin and hydroxychloroquine promotes CD8^+^ CTL and memory T-cell immune responses in tumor tissues via the controlled release of OVA and upregulation of MHC-I and CD86 costimulatory molecules in DCs [164]. To achieve the goal of targeting DCs more accurately, Rosalia et al. designed a PLGA-based CD-40-targeted cancer vaccine that showed significant enhancements in delaying tumor growth and extending the survival of tumor-bearing mice by facilitating antigen-specific antitumor CD8^+^ T-cell responses [165]. Recently, combination immunotherapy has become a particularly promising strategy for tumor treatment, and PLGA has been used to realize the codelivery of antiprogrammed cell death-1 (aPD1) and T-cell agonist (aOX40) agents to simultaneously rather than sequentially elicit the activation of T cells. These dual-immunotherapy NPs increased the ratio of CD8^+^ to regulatory T cells infiltrating the tumor, thereby promoting therapeutic efficacy in both B16F10 melanoma tumors and 4T1 breast cancers [166].

Indocyanine green (ICG) and imiquimod (R837) were coloaded by PLGA to achieve the eradication of preexisting tumors and enhance the antitumor immune response simultaneously. Moreover, with the combination of these particles and anti-CTLA-4, this strategy has been proven to delay tumor growth and extend survival in both 4T1 and CT26 tumor models [167]. PLGA polymers as biodegradable materials can also be combined with photothermal agents. Researchers produced anti-PD-1 peptide (AUNP12) and hollow gold nanoshell coencapsulated PLGA NPs, and these particles facilitated the effective inhibition of primary and distal tumor growth via an increasing percentage of CD8^+^ CTLs and secretion of IFN-γ [168]. In addition, the coadministration of anti-PD-1 peptide (AUNP12) and hollow gold nanoshell coencapsulated PLGA NPs with CpG has been proven to mediate the maturation of DCs in vitro and enable direct tumor necrosis in bilateral and lung metastatic 4T1 tumor-bearing mice [169]. The antibody-modified PLGA core was used to load the hydrophobic drug imatinib (IMT), which was developed as an inhibitor of tyrosine kinase and then manufactured as IR-780 and IMT codelivery PH-sensitive NPs, which showed a great capacity to stimulate an effective CD8^+^ T-cell antitumor immune response [170].

**Fig. 4:**
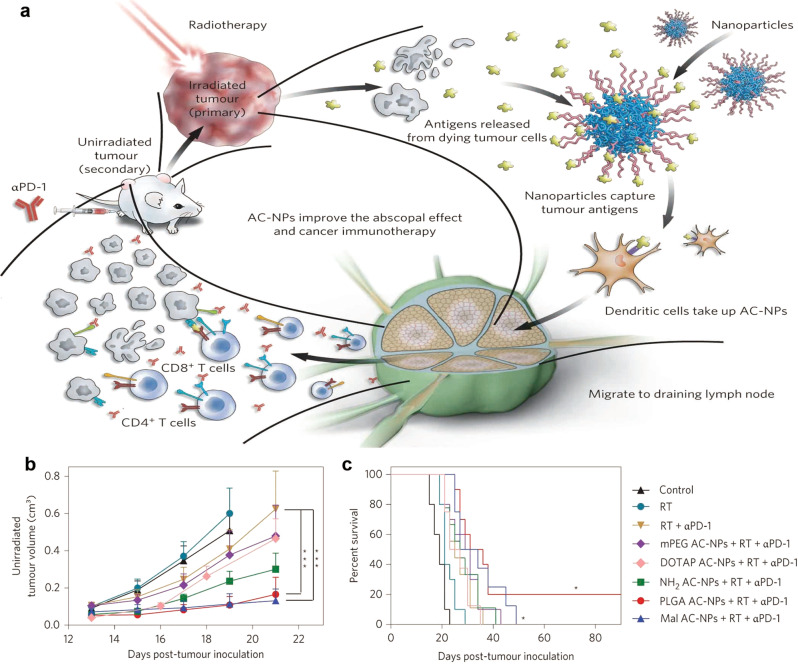
AC-NPs have the capacity to inhibit distant B16F10 xenografts. **a** Schematic illustration of cancer immunotherapy promotion by using antigen-capturing nanoparticles (AC-NPs) combined with radiotherapy and αPD-1 treatment. **b** Average tumor growth curves of abscopal tumors in mice treated with different administrations. **c** The survival rate of the treated mice in **b**. Reprinted with permission from [172]

Researchers have compared the capacity of synthetic long peptide-based cationic liposomes and PLGA NPs to induce an immune response. They proved that liposomes have advantages over PLGA particles for inducing the T-cell response. However, the mechanism of this phenomenon has not been investigated [171]. In addition, PLGA polymers used to formulate antigen-capturing nanoparticles (AC-NPs) were proven to promote an antitumor immune response and improve the efficacy of αPD-1 immunotherapy. The surfaces of PLGA NPs were modified by different chemical groups to bind tumor antigens; however, although all other AC-NPs except mPEG AC-NPs loaded plenty of proteins, PLGA and Mal AC-NPs showed a higher ability to improve the immunotherapeutic efficacy (Fig. 4) [172].

#### Hydrogels

Hydrogels can serve as antigen storage caverns because of their gelation properties, and they have been used as vectors to coload cytokines, proteins, DNAs, and so on [31, 173–179]. Alginate microparticle-based injectable gels were reported ~10 years ago and have been used to codeliver mature DCs and chemokines CCL21 and CCL19. The study showed that this hydrogel system could recruit host DCs to the injection site and migrate to local lymph nodes at the same time, thus providing a continual process to initiate the immune response [180]. Researchers have designed a two-step strategy to realize the recruitment of APCs and presentation of antigens via the injection of GM-CSF delivering mPEG−PLGA hydrogels followed by the administration of antigen-loaded vectors, which showed obvious antitumor immunotherapeutic potential [181]. Nanogel particles formed by cholesteryl pullulan showed the good function of delivering and cross-presenting antigens to medullary macrophages. In addition, the study revealed that this vaccine could significantly slow tumor growth with the help of Toll-like receptor agonists [182].

Hyaluronic acid–tyramine-based hydrogels have been used to deliver IFN-α to the injection site and to inhibit tumor proliferation via the coadministration of sorafenib [183]. In addition, a hyaluronic acid-pluronic F-127 hydrogel was used to prepare black phosphorus quantum dot nanovesicles (BPQD-CCNVs), GM-CSF, and LPS coloaded systems. The study showed that the sustained release of GM-CSF and LPS from the injection site could recruit and activate DCs. In addition, NIR irradiation combined with PD-1 antibody could generate a strong antitumor immune response [184].

Mooney and his colleagues manufactured an infection-mimicking system to coload GM-CSF, Toll-like receptor agonists (CpG-ODN) and a tumor lysate to achieve the recruitment and activation of DCs, which promoted a specific and effective antitumor immune response [185, 186]. The same team designed a cryogel-based delivery system to encapsulate GM-CSF and CpG-ODN. This vaccine could be subcutaneously injected into mice and controlled release immunomodulatory factors and cancer antigens, thus provoking strong antitumor T-cell responses and improving the survival rate of B16F10-bearing mice [187].

 Yang and coworkers reported that hydrogels formed by phosphatase enzymes had good potency in evoking humoral and cellular immune responses and could be used as protein vaccine adjuvants [188]. Moreover, they also proved that peptide Nap-GFFY hydrogels formed by a very simple process could also provoke a cogent CD8^+^ T-cell immune response [189]. Chao et al. designed a combination system in which ALG was cross-linked by multivalent cations and ^131^I-labeled catalase coadministered and jellified in a local tumor site, followed by systemic CTLA-4 injection. This strategy delayed local tumor growth and metastasis [190].

 Gu and coworkers designed a therapeutic scaffold formed by a ROS-responsive hydrogel to release gemcitabine (GEM) and an anti-PD-L1 blocking antibody (aPDL1) locally in tumor-bearing mice [191]. This system significantly decreased the level of ROS and the numbers of myeloid-derived suppressor cells and TAMs in the tumor site. Moreover, a 50% survival rate and 30% recurrence rate were observed in the aPDL1-GEM@Gel treatment group on account of primary and memory immune responses. In the same year, this research group also generated another immunotherapeutic gel for postsurgical tumor treatment, which was manufactured by mixing the fibrinogen solution containing anti-CD47 antibody-loaded CaCO_3_ NPs and thrombin solution in the postsurgical tumor site [192]. This strategy had the potency of preventing local and distant tumors via activating M1-type TAMs and promoting macrophage phagocytosis and antitumor immune responses.

**Fig. 5:**
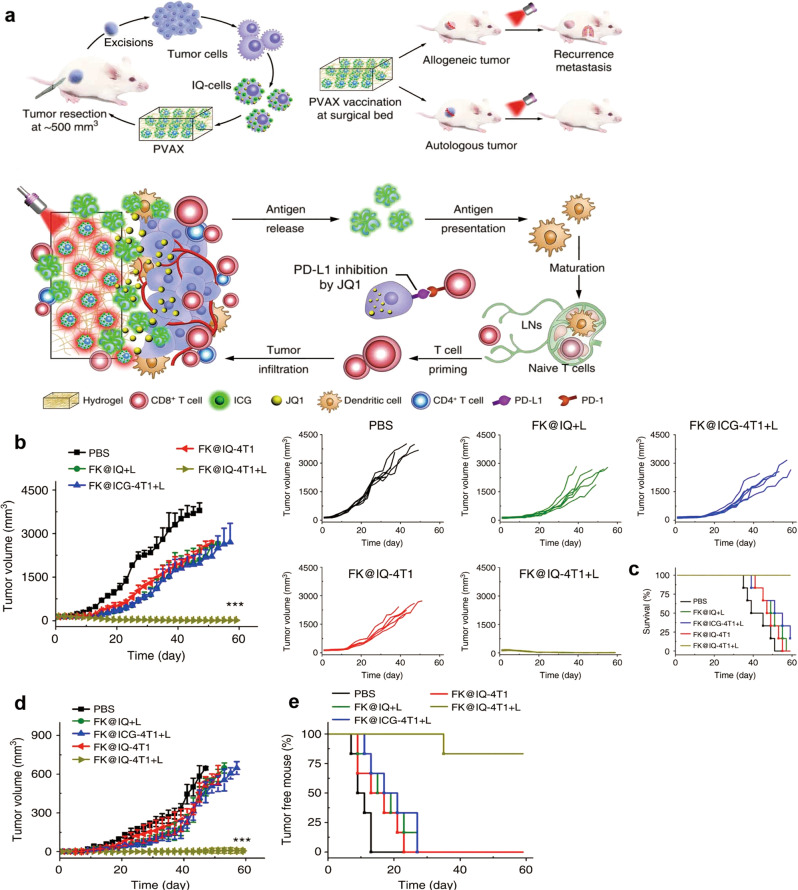
PVAX immunotherapy for both recurrent and metastatic 4T1 tumors. **a** Schematic depiction of the manufacture of PVAX for cancer immunotherapy. **b** Average and individual tumor growth curves of recurrent 4T1 xenografts in mice treated with different formulations. **c** Survival curves of the mice bearing 4T1 recurrent tumors. **d** Average tumor growth curves of the distant tumors treated with different formulations. **e** Tumor-free percentages of the abscopal tumor. Reprinted with permission from [195]

DNA-based supramolecular hydrogels were reported to recruit and activate APCs by releasing a high concentration of CpG, which could serve as a promising method for tumor immunotherapy [193]. Song et al. demonstrated a poly(*L*-valine) hydrogel for coencapsulating TCL, TLR3 agonist, poly(I:C) that realizes the controlled release of antigens and adjuvants, thus promoting antigen persistence and presentation to enhance the cytotoxic T-lymphocyte immune response against cancer [194]. A tumor-penetrable peptide-based hydrogel was prepared by encapsulating JQ-1 (a BRD4 inhibitor) and ICG coloaded tumor cells (Fig. 5) [195]. This vaccine could evoke a strong patient-specific immune response and prevent recurrence and metastasis of postsurgical tumors by NIR laser-triggered release of tumor-specific antigens and JQ-1.

### Inorganic biomaterials

#### Siliceous nanoparticles

**Fig. 6:**
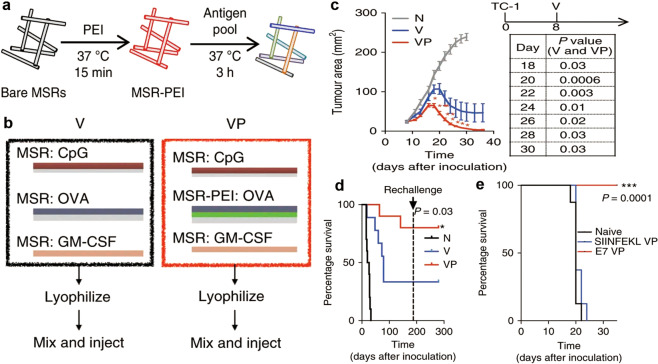
The MSR–PEI vaccine inhibits established tumors. **a** Schematic illustration of PEI and antigen adsorption. **b** Schematic depiction of the MSR vaccine and MSR–PEI vaccine. Tumor growth (**c**) and survival rate (**d**) of mice bearing E7-expressing TC-1 tumors rechallenged with TC-1 cells. **e** The survival rate of mice bearing E7-expressing TC-1 tumors treated with different formulations. Reprinted with permission from [220]

Mesoporous silica NPs can be prepared by using organosilane precursors to participate in hydrolysis and condensation reactions [196, 197]. Moreover, the surface of these particles can be modified with various reactive groups for different medical applications [198, 199]. Amino acid-modified silica NPs were reported to promote cytokine production, and silica nanospheres doped with Ca, Mg, and Zn (MS-Ca, MS-Mg, and MS-Zn) showed the capacity to provoke a Th1 anticancer immune response [200, 201]. Both spherical silica NPs and asymmetric mesoporous silica NPs were found to have the potential to activate and mature immune cells [202–206]. In addition, siliceous NPs also play a strong role in vaccine formulations such as the Japanese encephalitis vaccine [207], hepatitis B virus DNA vaccine [208], and oral hepatitis B vaccine [209], as well as in viral vaccine heat resistance [210] and viral inhibition [211]. Mesoporous silica-templated and hollow particles were designed to load antigens and adjuvants that showed a robust lymph node targeting and immune cell-activating capacity [212–216]. Yang et al. achieved the synthesis of dendritic mesoporous organosilica hollow spheres for the first time, which showed a significant potential to provoke an antitumor immune response [217]. Mooney and coworkers reported mesoporous silica rods (MSRs) with a high aspect ratio that spontaneously assembled as a macroporous structure to recruit DCs and generate humoral and cellular immune responses against tumors in the presence of GM-CSF and CpG [218]. They also modified these MSRs with PEG, PEG–RGD, or PEG–RDG groups [219] and PEI (Fig. 6) [220], which promoted immune cell activation and infiltration and may pave the way for cancer vaccination.

#### Iron oxide nanoparticles

Because iron oxide NPs have been approved for human use as MRI contrast agents and their degradation products are good for the body’s iron store, iron oxide NPs have been increasingly used simultaneously for cancer immunotherapy and imaging [221–223]. Iron oxide NPs can be modified with many cargos to improve the antitumor immune response, such as heat shock protein 70 (Hsp70) [224], R837 and Poly(I:C) [225], CpG-ODN [226], and ICG [227]. These NPs have the potential to realize the integration of imaging and therapy.

#### Gold nanoparticles

Photothermal immunotherapy is an effective treatment combining laser photophysical effects with immunoregulation [228–230]. Many photothermal biomaterials, such as gold nanorods [231], Prussian blue [232, 233], and NIR photosensitizers [234], have recently been used for cancer immunotherapy. Gold NP labeled melanoma-specific T cells show the potential to be noninvasively imaged by classical X-ray computed tomography, which provides a convenient way to track immune cells in immunotherapy [235]. More interestingly, gold NPs can predict the therapeutic response to immune checkpoint blockade after modification with programmed death-ligand 1 antibody (αPD-L1) [236]. Gold-based NPs may also be combined with adjuvants to promote antitumor immune responses and contribute to cancer immunotherapy [237, 238].

### Others

**Fig. 7:**
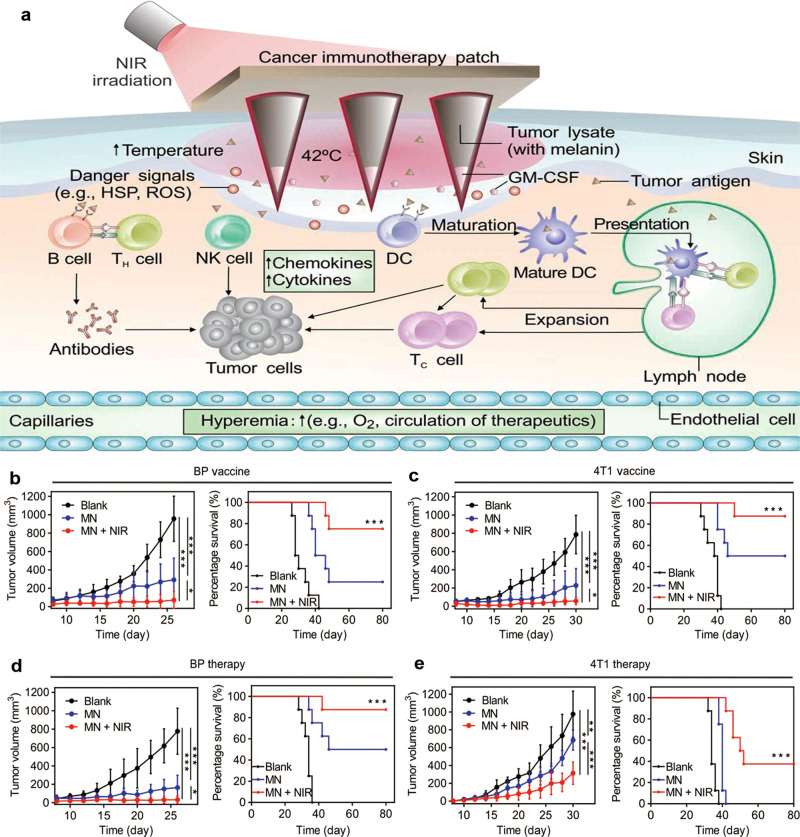
Local immunotherapy for various tumors via microneedles. **a** Schematic illustration of immunotherapy utilizing microneedles. **b** Average tumor growth and survival rate of treated C57BL/6J mice in the BP tumor model. **c** Average tumor growth and survival rate of treated BALB/c mice in the 4T1 tumor model. **d** Average tumor growth and survival rate of C57BL/6J mice in established BP tumor models. **e** Average tumor growth and survival rate of BALB/c mice in established 4T1 tumor models. Reprinted with permission from [255]

Microneedles with dimensions of <1 mm can be utilized to pierce the skin to the dermis in a minimally invasive and painless manner [239–241]. As a significant research target, microneedles have been used in many aspects, such as the delivery of small molecule and protein drugs and vaccines [242–244]. It is an inexpensive and convenient way to use microneedles for various medical applications [245–249]. There are many lymph nodes in the dermis; thus, microneedles achieve direct contact with DCs for antigen uptake and presentation [27]. To decrease the cost of treatment and reduce the dosage-dependent side effect [250–252] of immunomodulators, Gu and coworkers prepared biocompatible hyaluronic acid (HA)-based microneedles integrated with pH-sensitive dextran NPs containing aPD1 and glucose oxidase, which realized the substantial release of aPD1 and evoked a strong immune response in B16F10-bearing mice [253]. Meanwhile, the same group designed another microneedle system that combined aPD1 and 1-methyl-*DL*-tryptophan (1-MT), an inhibitor of IDO, to promote T-cell immunity and reduce immunosuppression [254]. Moreover, a hyaluronic acid-based MN encapsulated B16F10 melanoma whole tumor lysate and GM-CSF were proven to stimulate a robust antitumor immune response through spatiotemporal PTT and immunotherapy (Fig. 7) [255]. In addition to soluble microneedles, hollow microneedles can also be used for vaccine delivery and immunotherapy on account of the controllability and accuracy of the injection progress [256, 257]. Researchers compared four types of NP-loaded OVA and poly(I:C) by using hollow microneedles, which showed that PLGA NPs and liposomes could provoke stronger IgG2a responses [258]. In addition, a digitally controlled hollow microneedle system was used for the injection of liposomes containing an HPV E_743–63_ synthetic long peptide, thus reducing pain and the dosage of injection [259].

**Fig. 8:**
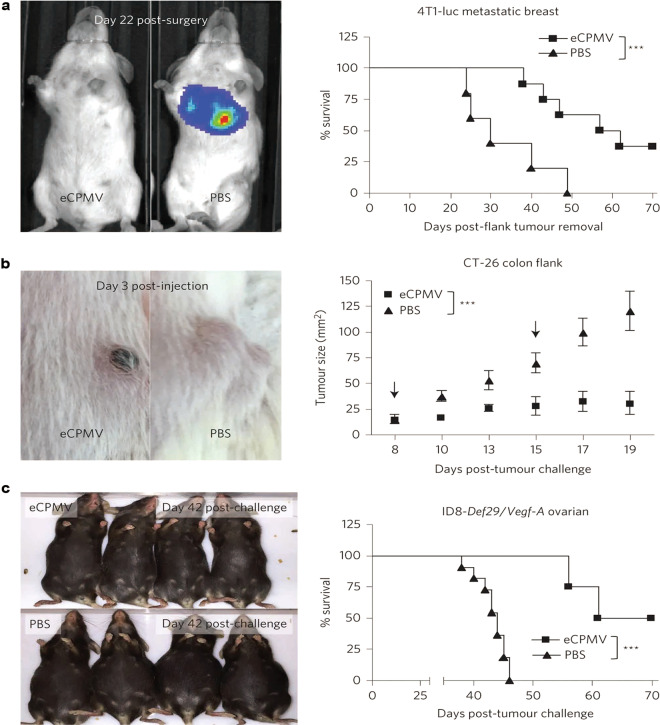
eCPMV immunotherapy for metastatic breast, colon, and ovarian tumors. **a** Photo and survival rate of mice in a metastatic breast tumor model. **b** Photo and survival rate of mice in a colon tumor model. **c** Photo and survival rate of mice with ID8-Defb29/Vegf-A ovarian cancer. Reprinted with permission from [267]

In recent years, biologically derived nanobiomaterials, such as cancer cell membranes, viral proteins, and DNAs, have started to be used for new cancer nanovaccines [260–263]. Researchers found that tumor antigens and subcellular particles in the cancer membrane, such as melanoma cells, could be loaded into various NPs, which induce specific cellular and humoral responses, thus preventing tumor growth [157, 264, 265]. Another approach is to form virus-like NPs via the self-assembly of viral proteins [266]. Furthermore, cowpea mosaic virus NPs effectively evoked cytokine secretion and inhibited tumor growth in various models (Fig. 8) [267].

## Conclusion

In this review, we have analyzed different strategies of immunotherapy and described advanced biomaterials that may be applied to improve therapeutic potency and reduce adverse effects. Although cancer immunotherapy is advancing at a high speed, the use of biomaterials to manufacture optimal systems for various tumors remains in its nascent stages. It is hoped that the biomaterials described in this review can be more widely and innovatively designed for cancer immunotherapy, thus promoting its efficacy and reducing immune-related side effects. Although preliminary advances have been made in the design of immunotherapy strategies based on biomaterials, many systems, including NPs, micelles, and hydrogels, can be loaded with multiple drugs and selected based on the targets identified in the patient’s biopsy sample. This personalized treatment will be an important and promising research direction in the future.
